# GLP-2 regulation of intestinal lipid handling

**DOI:** 10.3389/fphys.2024.1358625

**Published:** 2024-02-14

**Authors:** Kundanika Mukherjee, Changting Xiao

**Affiliations:** Department of Anatomy, Physiology and Pharmacology, College of Medicine, University of Saskatchewan, Saskatoon, SK, Canada

**Keywords:** glucagon-like peptide-2, neural pathway, intestine, chylomicron, triglyceride

## Abstract

Lipid handling in the intestine is important for maintaining energy homeostasis and overall health. Mishandling of lipids in the intestine contributes to dyslipidemia and atherosclerotic cardiovascular diseases. Despite advances in this field over the past few decades, significant gaps remain. The gut hormone glucagon-like peptide-2 (GLP-2) has been shown to play pleotropic roles in the regulation of lipid handling in the intestine. Of note, GLP-2 exhibits unique actions on post-prandial lipid absorption and post-absorptive release of intestinally stored lipids. This review aims to summarize current knowledge in how GLP-2 regulates lipid processing in the intestine. Elucidating the mechanisms of GLP-2 regulation of intestinal lipid handling not only improves our understanding of GLP-2 biology, but also provides insights into how lipids are processed in the intestine, which offers opportunities for developing novel strategies towards prevention and treatment of dyslipidemia and atherosclerotic cardiovascular diseases.

## Introduction

Balanced and regulated lipid metabolism is critical for whole-body energy homeostasis and overall health. In certain situations, lipid appearance (dietary lipid absorption in the intestine and lipoprotein production from the liver) is not balanced with its clearance, leading to abnormal levels and characteristics of lipids in the blood circulation (dyslipidemia) (Lewis et al., 2015). Dyslipidemia is common in patients with metabolic disorders (e.g., type 2 diabetes, obesity, and metabolic syndrome) and it increases the risk of atherosclerotic cardiovascular diseases (Lewis et al., 2015; Stahel et al., 2018). It is therefore important to understand the mechanisms of lipid metabolism for developing effective strategies for the prevention and treatment of atherosclerotic cardiovascular diseases.

Dietary lipids (mostly triglycerides, TGs) are processed in the intestine. TGs are absorbed into the intestinal absorptive cells (enterocytes) and packaged into either lipoprotein particles (chylomicrons, CMs) for secretion, or cytoplasmic lipid droplets (CLDs) for storage (Xiao et al., 2019a; Stahel et al., 2020; Zembroski et al., 2021; Ghanem et al., 2022). Following digestion in the small intestinal lumen, the digestion products (fatty acids and monoglycerides) are transported across the apical membrane of the enterocytes lining the villi of the intestine. Inside the enterocytes, they are resynthesized into TGs and form lipid droplets at the ER membrane. Most of these lipid droplets are directed to CM synthesis in the ER lumen where lipid-poor apolipoprotein B48 is lipidated to form pre-CMs. Pre-CMs are transported in transport vesicles to the Golgi apparatus for additional processing. Mature CM particles are exocytozed at the basolateral membrane, travel through the lamina propria, enter and transport through the lymphatics, and eventually join the blood circulation via the subclavian veins. CM biosynthesis, assembly and secretion in enterocytes have been extensively studied. It is well documented that CM production is subjected to regulation by nutrients, hormones and nutraceuticals and that CM production is increased in compromised metabolic status (Dash et al., 2015). Although the majority of dietary TGs undergo the CM synthesis and secretion route, some of the lipid droplets at the ER membrane are also used to form CLDs as a transient storage.

Besides immediate secretion of CMs following a meal, the intestine is also capable of retaining a significant portion of dietary lipids for secretion at later times. These two processes of lipid handling in the intestine, namely dietary lipid absorption and post-absorptive release of intestinally stored lipids, have been shown to be affected by various factors. Among these factors, gut hormones glucagon-like peptide-1 (GLP-1) and glucagon-like peptide-2 (GLP-2) have been shown to impact different aspects of these processes, as previously reviewed (Xiao et al., 2015; Stahel et al., 2021). Briefly, GLP-1 suppresses postprandial CM secretion by inhibiting CM biosynthesis and assembly. In contrast, GLP-2 stimulates CM secretion in both processes, the mechanisms of which remains elusive but begins to be better defined recently. This review aims to summarize current understanding of the mechanisms whereby GLP-2 modulates lipid handling in the intestine, with particular attention to emerging roles of neural pathways.

### Post-absorptive lipid storage and release in the intestine

Lipid storage and release in the intestine during post-absorptive state is a phenomenon with renewed recognition lately. Several earlier studies support prolonged lipid retention in the intestine into post-absorptive phase. In healthy, lean individuals, postprandial TG level in plasma rises rapidly, peaks at approximately 3–4 h after a lipid-rich meal, and gradually returns to fasting level after 6–8 h. However, ingestion of a second meal leads to a very early increase in plasma and CM TG, a phenomenon referred to as the “second-meal effect” (Jackson et al., 2001). This is attributed to the release of lipids stored in the intestine that are derived from the previous meal. In a stable isotope tracing study in humans, TGs from an earlier meal appeared in CM within as early as 20 min and up to 18 h after a second fat-rich meal (Chavez–Jauregui et al., 2010). Lipid storage may provide a significant portion of postprandial TG excursion. For example, regarding the second meal effects, it was estimated that ∼1/4 TG appearing during a morning meal is derived from the previous dinner (Chavez-Jauregui et al., 2010) and ∼1/3 of lunch lipids enter the blood after the onset of dinner (Jacome-Sosa et al., 2021). The exact location and form of lipid stores remain unclear, but CLDs in enterocytes and CMs in extra-enterocyte locations (such as intercellular space, lamina propria, and lymphatics) are likely (Xiao et al., 2019b; Xiao et al., 2020; Syed-Abdul et al., 2022a). In the small intestine, jejunum and duodenum retained abundant lipid droplets after fat ingestion in humans (Robertson et al., 2003; Xiao et al., 2018) and after oil gavage in mice (Hung et al., 2017). Besides a subsequent meal, several other factors have also been shown to release stored lipids from the intestine. Glucose ingestion in humans (Xiao et al., 2019a) or direct delivery of glucose into the upper small intestinal lumen in rats (Stahel et al., 2019) leads to CM output from the intestine. As shown in humans, increased CM output is accompanied by depletion of CLDs, pointing to the utilization of CLD TG as substrates for CM synthesis and secretion (Xiao et al., 2019a). Sham fat feeding (tasting without ingesting fat) similarly increases CM secretion (Chavez-Jauregui et al., 2010), the mechanism of which is not well understood but may invoke a neural circuitry involving taste receptors. The gut hormone GLP-2 has also been shown to cause rapid release of stored lipids from the intestine during post-absorptive state (discussed in more details below).

The physiological significance of intestinal lipid storage and release remains largely speculative. One possibility is that temporary retaining of part of dietary lipids in the intestine attenuates postprandial excursion of plasma TG. Rapid and complete secretion of dietary lipids into blood circulation would create a scenario where other organs may be overwhelmed with lipid overloading and lipotoxicity. In line with this, insulin resistant humans have reduced capacity of lipid storage in the intestine, which contributes to their postprandial lipemia (Jacome-Sosa et al., 2021). It is known that lipoprotein synthesis and secretion persist in fasting state. Post-absorptive release of stored lipids from the intestine may keep CM synthesis and secretory machinery ‘oiled’ and ready to ramp up with the next incoming meal (Xiao et al., 2020). It is unclear whether altered lipid retention/release in the intestine is the cause or consequence of pathophysiological conditions such as hyperlipidemic states, obesity, metabolic syndrome, and diabetes. Nonetheless, understanding the mechanism of this storage-release dynamics may offer unique opportunities for the prevention and treatment of dyslipidemia and atherosclerotic cardiovascular diseases.

### Glucagon-like peptide-2 (GLP-2)

GLP-2 was isolated and sequenced as a 33-amino acid peptide from human and porcine intestine. It belongs to the glucagon family of peptides, encoded by the proglucagon gene, that are majorly produced from the enteroendocrine cells of the intestine. The mammalian prohormone, proglucagon, undergoes tissue-specific posttranslational processing to give rise to GLP-2 in intestine and brain endocrine cells. GLP-2 is co-secreted with glucagon-like peptide-1 (GLP-1) in response to nutrients. Both these peptides are prone to degradation by dipeptidyl peptidase IV (DPPIV), a ubiquitous protease expressed in the intestine and vascular endothelium. Degradation of GLP-2 by DPPIV results in two circulating forms, GLP-2 (1-33) and GLP-2 (3-33).

#### Sources of GLP-2

GLP-2 is secreted by the enteroendocrine L-cells in distal small intestine and colon. It is a meal responsive hormone with its secretion stimulated by nutrients, mostly fat and glucose (Roberge and Brubaker, 1991; Brubaker and Anini, 2003). Besides intestinal L-cells, GLP-2 is also secreted by the neurons of brainstem (Vrang et al., 2007; 2008; Amato et al., 2016) that innervate the hypothalamic areas including paraventricular nucleus (PVN) and dorsomedial hypothalamus (DMH). GLP-2 immunoreactive fibres are also present in the arcuate nucleus (ARC) and PVN (Tang-Christensen et al., 2000). Since these hypothalamic areas are well-known for the regulation of food intake and energy balance, it is likely that GLP-2 may play important roles in energy balance as a neurotransmitter in these areas (Tang-Christensen et al., 2000).

#### Physiological functions of GLP-2

GLP-2 is well-known for regulating several physiological functions in the gastrointestinal tract. It was initially identified as an intestinotrophic factor for its promotion of small intestinal growth and repair. Other actions of GLP-2 in the gastrointestinal tract include stimulation of hexose transport and nutrient absorption, suppression of epithelial permeability, increase in mesenteric blood flow, improvement in intestinal barrier function, and reduction in gastrointestinal motility and acid secretion. These findings helped to identify GLP-2 as a possible therapeutic agent for the treatment of gastrointestinal diseases, such as short bowel syndrome (SBS), inflammatory bowel disease, and chemotherapy-induced mucositis. Besides the gastrointestinal tract, GLP-2 also acts centrally to regulate food intake and hepatic glucose metabolism.

#### Intestinotrophic effects of GLP-2

GLP-2 promotes intestinal growth and repair. Chronic administration of GLP-2 increased small intestinal weight and jejunal crypt-villus height in mice (Drucker et al., 1996; Brubaker et al., 1997). Chronic treatment with GLP-2 also increased villus height and crypt depth in short bowel jejunostomy patients (Jeppesen et al., 2001; 2009). These studies identified GLP-2 as a growth-promoting factor that stimulates intestinal growth and repair and supported the development of GLP-2 as a treatment for SBS. Teduglutide, a DPPIV resistant GLP-2 analogue with a prolonged half-life (∼3–5 h) compared to native GLP-2 (∼7 min), was approved for the treatment of SBS in 2012. Long-acting GLP-2 analogs, such as apraglutide (half-life 72 h), also exhibit improved efficacy in promoting intestinal growth (Hargrove et al., 2020). Mechanistically, GLP-2 inhibits crypt and enterocyte apoptosis and stimulates crypt cell proliferation, leading to expansion of the mucosal epithelium and increased mucosal surface area (Drucker, 2002), via activation of ErbB signaling (Yusta et al., 2009) and growth factors like insulin-like growth factor-1 (Murali et al., 2012).

#### GLP-2 in nutrient absorption

GLP-2 increases nutrient absorption. GLP-2 infusion increased glucose and amino acid uptake in total parenteral nutrition-fed piglets (Guan et al., 2003). GLP-2 can increase glucose uptake indirectly by increasing glucagon secretion as GLP-2 receptor (GLP-2R) is expressed in pancreatic alpha cells (De Heer et al., 2007) or through portal drained visceral blood (Guan et al., 2003). Exogenous GLP-2 administration increased absorption of intestine luminal fatty acids (Hsieh et al., 2009). SBS patients suffer from poor nutrient absorption and may require total parenteral nutrition. Administration of GLP-2 or its analogue in these patients improved their overall energy, carbohydrate, fluid and electrolyte absorption (Jeppesen et al., 2001; 2009; Eliasson et al., 2021).

#### GLP-2 in food intake and gut motility

GLP-2 suppresses food intake and gastric emptying in humans and rodents. Administration of GLP-2 in the brain (intracerebroventricular) decreased food intake in mice (Guan et al., 2012) and rats (Tang-Christensen et al., 2000) and reduced gastrointestinal motility in mice (Guan et al., 2012). These effects require the activation of GLP-2R in proopiomelanocortin (POMC) neurons in the hypothalamus (Guan et al., 2012). Interestingly, GLP-2 actions on food intake were abolished in rats with the loss of GLP-1R (Tang-Christensen et al., 2000). On the contrary, central inhibitory actions of GLP-2 on food intake in mice is increased after loss of Glp-1r signaling (Lovshin et al., 2001). It remains controversy regarding the interplay between GLP-1 and GLP-2 on food intake. In fasted rats, GLP-2 administration into the nucleus tractus solitarius (NTS), where Glp-2r is expressed (Lovshin et al., 2004), resulted in inhibition of food intake, which was abolished by blockade of melanocortin 4 receptor (MC4R) (Sun et al., 2021). These findings indicate that GLP-2 regulates food intake via a central mechanism with GLP-2R and MC4R as important mediators.

Gastric emptying is an essential process in regulating short-term food intake. Intravenous GLP-2 infusion decreases gastric emptying in pigs (Wøjdemann et al., 1998) and humans (Nagell et al., 2004). It has been shown that GLP-2 increases murine gastric capacity by inhibiting gastric fundic tone (Amato et al., 2009). This effect seems to be mediated by vasoactive intestinal peptide (VIP), as VIP receptor (VIPR) desensitization reduced gastric relaxation induced by GLP-2 (Amato et al., 2009). The increased gastric capacity may underline the short-term inhibition of food intake by GLP-2 (Baccari et al., 2022).

#### Anti-inflammatory function of GLP-2

Anti-inflammatory function of GLP-2 has been shown in various studies. GLP-2R activation reduced the expression of macrophage-dependent cytokines and LPS-induced inflammation in human islets (He et al., 2021). In pigs, chronic administration of GLP-2 also reduced LPS-induced inflammation (Qi et al., 2015). Necrotizing enterocolitis is a severe gastrointestinal disorder in premature infants. In experimental rat model of necrotizing enterocolitis, chronic GLP-2 administration decreased mucosal inflammatory cytokine production (Nakame et al., 2016). Chronic administration of Glp-2r antagonist improved hepatic inflammation in obese mice (Cani et al., 2009). GLP-2 reduced hepatic inflammation and fibrosis in multidrug resistance 2 knockout mice by inactivating hepatic stellate cells and activating intestinal Farnesoid X receptor signaling (Fuchs et al., 2023), while loss of Glp-2r signaling in mice activated hepatic stellate cells and exacerbated diet-induced steatohepatitis (Fuchs et al., 2020). GLP-2 treatment also reduced pro-inflammatory cytokines and crypt cell apoptosis in rats with intestinal inflammation (Sigalet et al., 2007). These anti-inflammatory effects of GLP-2 are mediated by activation of VIP in enteric neurons (Sigalet et al., 2007), highlighting an important role of the enteric neural pathway in this action.

#### Regulation of blood flow by GLP-2

GLP-2 increases intestinal blood flow in healthy humans (Bremholm et al., 2009; Xiao et al., 2019c) and in patients with SBS (Bremholm et al., 2011). Its enhancement in intestinal blood flow is nitric oxide (NO) dependent, as co-infusion with nitric oxide synthase (NOS) inhibitors attenuated this effect in pigs (Guan et al., 2003), rats (Deniz et al., 2007) and humans (Xiao et al., 2019c). GLP-2R is expressed on enteric neurons expressing VIP and endothelial NOS (Guan et al., 2006). Both VIP and endothelial NOS are well-known for regulating mucosal blood flow. These vasoactive neurotransmitters in the enteric neurons therefore are important mediators in the increase in blood flow by GLP-2.

#### GLP-2R expression

GLP-2 actions require binding and activating its receptor, GLP-2R, a G-protein coupled receptor and a member of the glucagon-secretin receptor family. In humans and rodents, GLP-2R is predominantly expressed in the gastrointestinal tract and central nervous system. In the intestine, GLP-2R mRNA transcripts are abundant in the lamina propria of the mucosa layer, in the circular and longitudinal muscle layers, and in the nerve plexuses within the duodenum, and the mucosa and nerve plexuses of the jejunum and ileum (Wismann et al., 2017). Regarding specific cell types, GLP-2R is expressed on myofibroblasts (Ørskov et al., 2005), enteroendocrine cells (Yusta et al., 2000) and enteric neurons (Bjerknes and Cheng, 2001; Guan et al., 2006). Notably, enterocytes do not express GLP-2R (Yusta et al., 2000; 2019; Nelson et al., 2007; Pedersen et al., 2015). GLP-2R is also expressed in the nodose ganglia of vagus nerve (Nelson et al., 2007; Amato et al., 2016), which contains the cell bodies of vagal afferent nerve fibers. The physiological significance of GLP-2R expression in anatomical locations and specific cell types in the intestine has yet to be determined. Centrally, GLP-2R is mostly expressed in DMH and ARC of the hypothalamus and is also located in the brainstem (dorsal motor nucleus of vagus nerve [DMV]) and hippocampus (parabrachial neurons) (Guan, 2014). Since these are major energy balance regulating areas of the brain, it is conceivable that GLP-2 plays important roles in metabolic regulation.

#### GLP-2 in metabolic disorders

Elevated levels of circulating GLP-2 have been reported in streptozotocin-induced diabetic rats compared with nondiabetic controls (Fischer et al., 1997). Obese subjects also have elevated plasma GLP-2 levels in both fasting and postprandial states, which correlated with increases in hemoglobin A1c and insulin resistance (Verdam et al., 2011; Geloneze et al., 2013). Postprandial plasma GLP-2 level was found to be unaltered in obese subjects compared to normal weight individuals following a fat meal (Higgins et al., 2020). In obese insulin-resistant subjects, postprandial secretion of GLP-2 was blunted (Higgins et al., 2020). Further, circulating levels of GLP-2 are increased in obese subjects following bariatric surgery (Cazzo et al., 2016). Normal GLP-2R signaling may be protective against dysregulated lipid metabolism. In hamsters, GLP-2 actions on increasing plasma TG in insulin resistant states may contribute to postprandial dyslipidemia (Hein et al., 2013). In addition, chronic treatment with a GLP-2R antagonist, GLP-2 (3-33), exacerbated insulin resistance in high-fat fed mice (Baldassano et al., 2015) and hepatic lipid accumulation (Baldassano et al., 2016). On the contrary, high fat-fed GLP-2R knockout mice had reduced hepatic lipoprotein (very-low-density lipoprotein) secretion and similar fasting plasma TG levels as compared to chow-fed mice (Taher et al., 2018). Chronic intraperitoneal GLP-2 administration stimulated very-low-density lipoprotein secretion and increased fasting plasma TG levels in chow-fed but not high fat-fed hamsters (Taher et al., 2018). Chronic administration of GLP-2 reduced inflammation in the brain of obese mice (Nuzzo et al., 2019). Loss of Glp-2r in mice reduces atherosclerosis (Kahles et al., 2023). Finally, patients with myocardial infarction have higher circulating levels of GLP-2, making GLP-2 an early indicator for cardiovascular diseases (Kahles et al., 2023). Although these studies suggest beneficial effects of endogenous GLP-2 against metabolic disorders, it remains unclear whether increased GLP-2 secretion and action is pathological for or a characteristic of metabolic disorders.

### Regulation of lipid handling in the intestine by GLP-2

Besides the above-mentioned biological functions, GLP-2 has been shown to play pleiotropic roles in regulating lipid handling in the intestine. Considering the unique aspects of GLP-2’s actions in this regard, it is important to make distinctions of its effects on two separate processes, i.e., dietary fat absorption during postprandial state, and the release of intestinally stored lipids during post-absorptive state.

#### Lipid absorption

GLP-2 enhances lipid absorption in the intestine. In healthy humans, intravenous infusion of GLP-2 increased plasma levels of free fatty acids and TG during meal ingestion, indicating enhanced absorption of luminal lipids (Meier et al., 2006). In mice and hamsters, acute GLP-2 administration enhanced lipid absorption and CM secretion during oral oil gavage (Hsieh et al., 2009). GLP-1, another gut hormone co-secreted with GLP-2 in a 1:1 M ratio from enteroendocrine L cells, decreased lipid absorption in hamsters (Hein et al., 2013). However, when GLP-1 and GLP-2 were co-infused for short-term (30 min), there was increased lipid absorption, suggesting a predominant effect of GLP-2 (Hein et al., 2013). In contrast, prolonged (120 min) co-infusion of GLP-1 and GLP-2 decreased lipid absorption. In addition, inhibition of DPPIV, the enzyme that cleavages GLP-1 and GLP-2, decreased lipid absorption (Hein et al., 2013). This suggests that under normal physiological conditions the actions of GLP-2 predominate to enhance lipid absorption, which is lost under conditions of sustained GLP-1 activity. Overall, studies in humans and rodents show that exogenous GLP-2 enhances dietary lipid absorption, although the physiological significance of this has yet to be determined.

#### Lipid mobilization

As mentioned above, the intestine withholds a portion of ingested dietary lipids into the post-absorptive state. A unique feature of GLP-2 in intestinal lipid handling is its post-absorptive mobilization of such intestinally stored lipids. In humans, administration of GLP-2 7 h after a fat load increased plasma and CM TG (Dash et al., 2014). The increased CM secretion following GLP-2 is attributed to the release of CM that are “pre-formed” (i.e., not newly synthesized) and reside in locations outside enterocytes, such as intercellular space, lamina propria and mesenteric lymphatics. Several evidence support this notion. Firstly, tracer kinetics data and mathematic modelling did not support enhanced synthesis of new apolipoprotein B48 (apoB48, the structural apolipoprotein on CM); instead, they pointed to increased appearance of apoB48 in blood circulation without the contribution of new apoB48 synthesis. In line with this, GLP-2 did not affect CM biosynthesis pathway in humans (Syed-Abdul et al., 2022b) and CM particle size in lymph fluid in rats (Stahel et al., 2019). Secondly, retinal palmate tracing of dietary lipids supported that the increased TG in plasma and CM originated from the earlier meal (Dash et al., 2014). Mobilization of intestinal lipid stores by GLP-2 during fasting was confirmed in studies with rodents, including mice and hamsters (Hsieh et al., 2015) and rats (Stahel et al., 2019).

#### Mechanisms of GLP regulation of intestinal lipid handling

The exact mechanisms whereby GLP-2 modulates intestinal lipid handling remains elusive. In the following sections, we summarize direct and indirect evidence that help provide mechanistic insights for both processes of lipid handling.

#### Postprandial lipid absorption

Several mechanisms have been proposed for GLP-2’s enhancement in postprandial lipid absorption. In mice and hamsters, GLP-2 enhances dietary lipid absorption via glycosylation of CD36 (Hsieh et al., 2009). CD36 is a scavenger receptor mediating the transport of fatty acids across the plasma membrane of various cell types, including enterocytes (Nassir et al., 2007). CD36 glycosylation by GLP-2 may provide functional enhancement in fatty acid uptake by the enterocytes. How GLP-2 increases CD36 glycosylation is unknown.

Since GLP-2R is not expressed on the enterocytes where CM biosynthesis occurs, enhanced lipid absorption is likely indirect. One possibility is via GLP-2’s effects on the secretion of several hormones, as GLP-2 infusion in humans inhibits ghrelin (Banasch et al., 2006) and stimulates glucagon (Meier et al., 2006) secretion. GLP-2 also stimulates VIP secretion from enteric VIP-expressing neurons (de Heuvel et al., 2012). Enterocytes are known to express VIP receptor (Dharmsathaphorn et al., 1983) and VIP may activate VIPR1 on enterocytes to stimulate NO production (Spessert, 1993; González et al., 1997). As discussed above, GLP-2 exerts anti-inflammatory effects via activation of VIP neurons (Sigalet et al., 2007). In a recent study, it was shown that intestine luminal lipids stimulate VIP-expressing neurons to release VIP, which activates VIPR2 on type-3 innate lymphoid cells to release IL-22 and subsequently IL-22 stimulates enterocytes to enhance lipid absorption (Talbot et al., 2020). It is therefore likely VIP-neurons are an important intermediate cell type that responds to GLP-2 stimulation by secreting VIP to enhance lipid absorption. This intriguing hypothesis remains to be tested in future studies.

Nitric oxide (NO) signaling was suggested to mediate enhanced postprandial lipid absorption by GLP-2. GLP-2-stimulated lipid absorption and CM secretion was blocked by NOS inhibitor in hamsters and endothelial NOS-deficient mice were resistant to GLP-2 stimulation in CM secretion (Hsieh et al., 2015). GLP-2R is expressed on NOS-positive cells (Cinci et al., 2011); therefore, it is conceivable that GLP-2 enhances lipid absorption through stimulating NO production. Importantly, the NO donor S-nitroso-L-glutathione stimulated CM production *in vitro* in primary enterocytes (Hsieh et al., 2015). This indicates that NO production in enterocytes can have a direct effect on lipid absorption. VEGF-C receptor (VEGFR3) signaling is required for lipid absorption along with NO production (Shew et al., 2018). VEGF is released by myofibroblasts which express GLP-2R and VEGF can activate its receptor on the enterocytes (Siafakas et al., 1999). It is possible that a GLP-2-VEGF-NO pathway may be operative for the stimulation of lipid absorption. Several of GLP-2’s other actions are underlined by its stimulation on NO production, which may indirectly contribute to GLP-2’s effects on lipid absorption. For instance, GLP-2-stimulated increase in intestinal blood flow in pigs was blunted by NOS inhibitor (Guan et al., 2003). NO is a vasodilator, thus increased NO production may lead to increased blood flow. Increased mesenteric blood flow, secondary to increased NO production, might contribute to increased CM secretion by GLP-2; however, a direct link between blood flow and CM secretion has not been established. Local NO production by specific cells may play important roles in mediating GLP-2’s effects on intestinal lipid absorption. In a recent study, Grande et al. showed that GLP-2 stimulates dietary lipid absorption and CM production in mice and hamsters via neuronal NOS (nNOS) (Grande et al., 2022). Specifically, loss of nNOS in hamsters and mice ablated GLP-2 enhancement in lipid absorption. The exact nature of this pathway remains unclear, but protein kinase G (PKG) seems to be downstream of nNOS, thus GLP-2 invokes a nNOS-PKG-dependent pathway (Grande et al., 2022).

#### Post-absorptive release of lipid stores

NO signaling has been proposed to mediate GLP-2’s effects on postprandial release of intestinal lipid stores. In hamsters, the NO donor S-nitroso-L-glutathione stimulated, while NOS inhibitor attenuated, the release of stored TGs during post-absorptive stage (Hsieh et al., 2015). In humans, co-infusion of GLP-2 and a NOS inhibitor attenuated GLP-2’s effects on stimulating mesenteric blood flow but did not affect its effects on stimulating post-absorptive CM release (Xiao et al., 2019b). The discrepancy may be due to species differences. It is also possible that NO production from specific cell types in the intestinal region, but not systemic NO production, mediates GLP-2’s effects on post-absorptive release of intestinally stored lipids. If this being the case, the specific cell type(s) remain undefined. As discussed above, neuronal NOS was shown to underly GLP-2’s enhancement in postprandial lipid absorption (Grande et al., 2022). Whether GLP-2 mobilizes intestinal lipid stores via neuronal NOS remains to be studied.

An additional mechanism for GLP-2 to release intestinal lipid stores may involve the modulation of lymphatic functions. Following secretion from the enterocytes, CMs enter the lacteals and transport in the mesenteric lymph ducts before joining the blood circulation. VEGF signaling plays important roles in regulating the contractility, pumping and opening/closing of the lymphatic endothelial wall (Breslin et al., 2007; Choe et al., 2015; Zhang et al., 2018). VEGF, which is expressed on the smooth muscle cells lining the lacteal, is vital for sustained increase in lymphatic contraction and lipid absorption (Syed-Abdul et al., 2022a). It has been shown that GLP-2 promotes intestinal growth through VEGF release from subepithelial myofibroblasts (Bulut et al., 2008). It is possible that increased VEGF release in response to GLP-2 enhances lymphatic functions to promote lipid output. CD36 expression on lymphatic endothelial cells increases from lacteals to collecting vessels and is responsible for maintaining lymphatic integrity and lipid absorption (Cifarelli et al., 2021). An intriguing hypothesis is that VEGF and CD36 are downstream mediators of GLP-2 in mobilizing intestinal lipid stores by regulating lymphatic functions.

#### Potential neural pathways in mediating GLP-2’s effects on intestinal lipids

GLP-2R is expressed on neuronal cells in both the intestine and the brain. In the intestine, it is expressed on the enteric neurons (Bjerknes and Cheng, 2001; Guan et al., 2006). GLP-2R activation on enteric neurons contributes to GLP-2 promotion of intestinal growth and repair (Bjerknes and Cheng, 2001). Centrally, GLP-2R is expressed mostly in the energy balance regulating areas of the brain and several of these areas are innervated by GLP-2 immunoreactive terminal fibres from the brainstem (Larsen et al., 1997; Yusta et al., 2000; Lovshin et al., 2001). CNS GLP-2R signaling has been shown to play significant roles in regulating several physiological processes, including feeding behavior and gastrointestinal function. Chronic intracerebroventricular infusion of GLP-2 suppressed food intake and increased POMC mRNA in the ARC (Guan et al., 2012). POMC neurons are well-known for regulating energy balance by integrating long-term adiposity and short-term satiety endocrine signals. POMC specific Glp-2r knockout in mice increased food intake and gastric motility (Guan et al., 2012). CNS GLP-2R signaling has also been shown to regulate peripheral metabolism, thus POMC-specific Glp-2r knockout in mice impaired whole-body glucose metabolism and increased hepatic glucose production (Shi et al., 2013).

The importance of central GLP-2/GLP-2R is also highlighted by its link to behavioral and neuropathological conditions. Astrocytes are non-neuronal cells that are abundantly present in the CNS. They are important for homeostasis, defence and regeneration of the CNS and active contribution to pathogenesis of neurodegenerative disorders including Alzheimer’s disease. GLP-2 increased proliferation of cultured rat astrocytes (Velázquez et al., 2003). This is in line with higher expression of GLP-2R in younger passages of astrocyte cell culture (with higher capacity of proliferation) compared to older passages of the culture (Velázquez et al., 2022). GLP-2 has also been shown to restore memory and neurogenesis in experimental Alzheimer’s disease mouse model (Sasaki-Hamada et al., 2019). Thus, targeting GLP-2/GLP-2R signaling may be beneficial for the treatment of Alzheimer’s disease. GLP-2 analogue exhibited neuroprotective properties against Parkinson’s disease (Su et al., 2021; Zhang et al., 2021). Central GLP-2 infusion also showed prevention and protection against inflammation-induced memory impairment and anxiety in mice (Iwai et al., 2015).

Intestinal and brain neuronal GLP-2R expression and the regulation of feeding behavior and hepatic glucose metabolism by central GLP-2 signaling strongly suggest a neural network in regulating its effects on intestinal lipid handling. Besides evidence that local neural pathways are involved in GLP-2 enhancement of lipid absorption, post-absorptive mobilization of intestinal lipid stores also involves a neural mechanism. In a recent study, we demonstrated that the full effects of GLP-2 in releasing lipid stores during post-absorptive state requires a neural pathway involving the CNS (Mukherjee et al., 2023). In consistency with previous studies, intraperitoneal administration of GLP-2 during post-prandial period stimulated intestinal lipid output in rats. This was accompanied with activation of POMC neurons in the ARC of hypothalamus. When the gut-brain neural communication was disrupted with subdiaphragmatic vagotomy, GLP-2’s effects on intestinal lipid release was blunted. This supports that GLP-2 mobilizes lipid storage in the intestine through both local and central mechanisms. The exact nature of this pathway remains to be defined. MC4R signaling in the hypothalamus is activated by POMC neurons to control feeding and gastric emptying in rats (Guan et al., 2012; Guan, 2014; Sun et al., 2021). GLP- 2R activation in POMC neurons increases vagal outflow by activating MC4R in the brainstem (Guan et al., 2012; Shi et al., 2013). In light of their roles in regulating feeding behavior and hepatic glucose metabolism, CNS GLP-2R and MC4R are likely candidate key players along this pathway. Collectively, a working model for GLP-2 mobilization of lipid stores in the intestine involves both peripheral and central mechanisms, the latter requiring further elucidation.

## Concluding remarks and future directions

**FIGURE 1:**
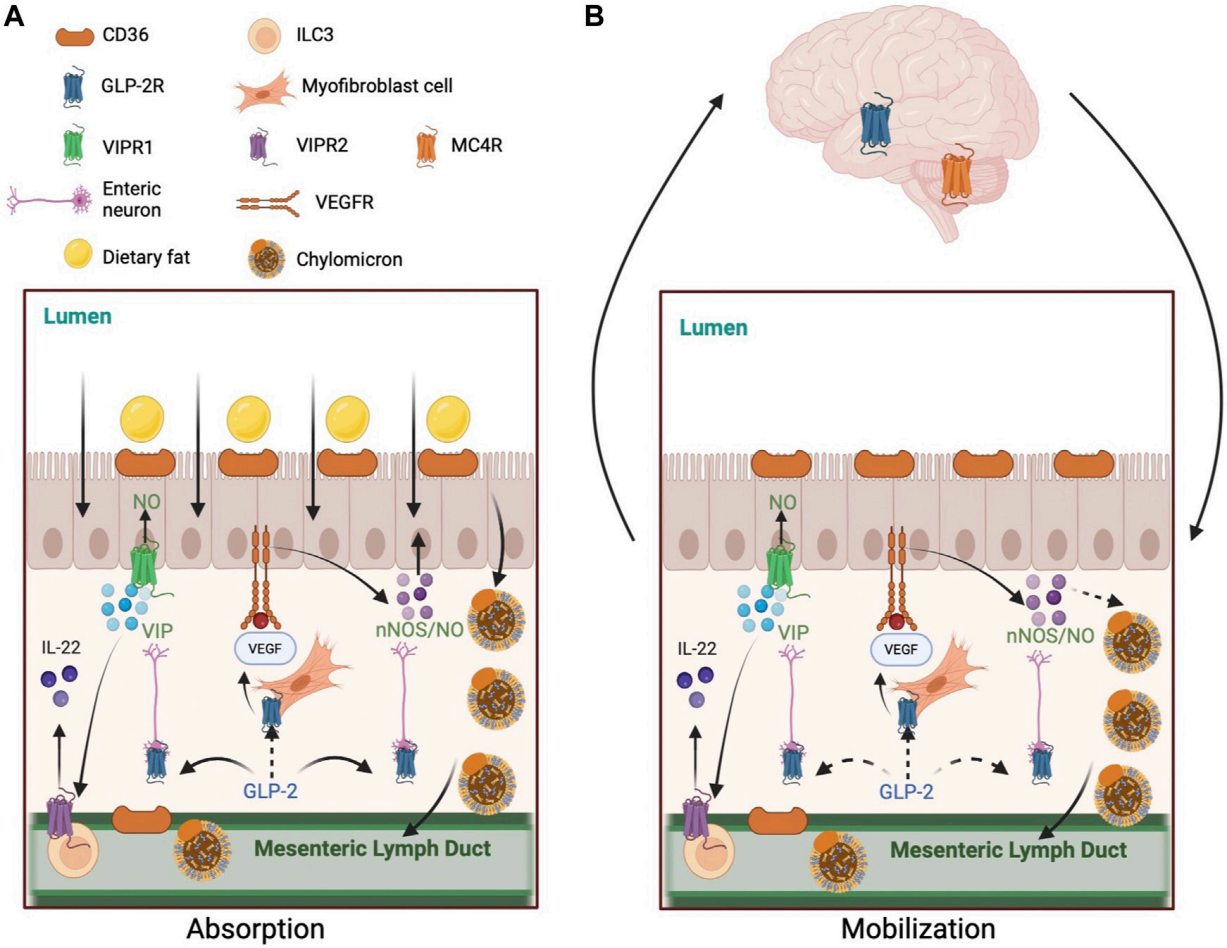
Mechanisms of GLP-2 regulation of intestinal lipid handling. **(A)** GLP-2 enhances lipid absorption during postprandial state. 1) GLP-2 increases CD36 glycosylation on the apical membrane of enterocytes. 2) GLP-2 stimulates NO production in NOS-expressing cells, including enterocytes, endothelial cells, and enteric neurons. GLP-2 activates neuronal NOS which subsequently activates protein kinase G. GLP-2 may stimulate VEGF release by myofibroblast to activate VEGFR on enterocytes to enhance lipid absorption directly or indirectly via NO production. GLP-2 increases intestinal blood flow via increased NO production, which may indirectly enhance lipid absorption. 3) GLP-2 stimulates VIP production by enteric neurons. VIP may activate VIPR1 on enterocytes to stimulate NO production and lipid absorption. VIP may also activate VIPR2 on type-3 innate lymphoid cells to release IL-22, which activates IL-22 receptors on enterocytes to enhance lipid absorption. **(B)** GLP-2 promotes the release of intestinally stored lipids during post-absorptive state. 1) GLP-2 stimulated NO production mediates the mobilization of intestinal lipid stores in rodents, but not humans. GLP-2 may stimulate VEGF release by myofibroblast to activate VEGFR on enterocytes to enhance lipid mobilization directly or indirectly via NO production. 2) GLP-2 may enhance lymphatic function by stimulating VEGF release from subepithelial myofibroblasts and longitudinal and circular muscles surrounding the lymphatics. CD36 on lymphatic endothelial cells may respond to GLP-2. 3) A neural pathway involving the CNS also participates GLP-2 mobilization of intestinal lipid stores. This pathway may include GLP-2 activation of its receptors on vagal afferent neurons, central activation of GLP-2R and MC4R, and vagal efferent outflow to the intestine. Solid arrows indicate known pathways. Dashed arrows indicate implicated but not yet elucidated pathways. Abbreviations: CD36, cluster of differentiation 36; GLP-2, glucagon-like peptide-2; GLP-2R, glucagon-like peptide-2 receptor; IL-22, interleukin 22; MC4R, melanocortin 4 receptor; NO, nitric oxide; NOS, nitric oxide synthase; VEGF, vascular endothelial growth factor; VEGFR, vascular endothelial growth factor receptor; VIP, vasoactive intestinal peptide; VIPR, vasoactive intestinal peptide receptor.

The intestine is a key organ for lipid handling. Beside the well-recognized role in dietary lipid absorption, it is also increasingly recognized that the intestine is capable of retaining a releasable pool of lipids during post-absorptive period. The regulation of these processes, despite significant advances, remain not fully understood. GLP-2, a gut hormone with a range of biological roles, regulates lipid handling in the intestine, both during dietary lipid absorption and during post-absorptive release of stored lipids. How GLP-2 mediates each of these two processes are being elucidated. However, the exact mechanisms are not fully defined. The current knowledge is that GLP-2 “indirectly” enhances lipid absorption via intermediate GLP-2R-expressing cell(s), that neural pathways are invoked by GLP-2 at least partly in both processes, and that both local and central regulatory mechanisms are likely involved (Figure 1). It is hoped that better understanding of the mechanism whereby GLP-2 regulates lipid handling in the intestine will provide health benefits beyond its current clinical use for the treatment of short-bowel syndrome.

## Abbreviations

ARC, arcuate nucleus; CD36, cluster of differentiation 36; CLD, cytoplasmic lipid droplet; CM, chylomicron; DMH, dorsomedial hypothalamus; DPPIV, dipeptidyl peptidase IV; GLP-2, glucagon-like peptide-2; GLP-2R, glucagon-like peptide-2 receptor; IL-22, interleukin 22; MC4R, melanocortin 4 receptor; NO, nitric oxide; NOS, nitric oxide synthase; NTS, nucleus tractus solitarius; POMC, proopiomelanocortin; PVN, paraventricular nucleus; SBS, short bowel syndrome; TG, triglyceride; VEGF, vascular endothelial growth factor; VEGFR, vascular endothelial growth factor receptor; VIP, vasoactive intestinal peptide; VIPR, vasoactive intestinal peptide receptor.

## References

[B1] Amato A. Baldassano S. Mulè F. (2016). GLP2: an underestimated signal for improving glycaemic control and insulin sensitivity. J. Endocrinol. 229, R57–R66. 10.1530/JOE-16-0035 27048234

[B2] Amato A. Baldassano S. Serio R. Mulè F. (2009). Glucagon-like peptide-2 relaxes mouse stomach through vasoactive intestinal peptide release. Am. J. Physiology-Gastrointestinal Liver Physiology 296, G678–G684. 10.1152/ajpgi.90587.2008 19109404

[B3] Baccari M. C. Vannucchi M. G. Idrizaj E. (2022). Glucagon-like peptide-2 in the control of gastrointestinal motility: physiological implications. Curr. Protein Pept. Sci. 23, 61–69. 10.2174/1389203723666220217142935 35176986

[B4] Baldassano S. Amato A. Caldara G. F. Mulè F. (2016). Glucagon-like peptide-2 treatment improves glucose dysmetabolism in mice fed a high-fat diet. Endocrine 54, 648–656. 10.1007/s12020-016-0871-3 26832341

[B5] Baldassano S. Rappa F. Amato A. Cappello F. Mulè F. (2015). GLP-2 as beneficial factor in the glucose homeostasis in mice fed a high fat diet. J. Cell Physiol. 230, 3029–3036. 10.1002/jcp.25039 25967277

[B6] Banasch M. Bulut K. Hagemann D. Schrader H. Holst J. J. Schmidt W. E. (2006). Glucagon-like peptide 2 inhibits ghrelin secretion in humans. Regul. Pept. 137, 173–178. 10.1016/j.regpep.2006.07.009 16928403

[B7] Bjerknes M. Cheng H. (2001). Modulation of specific intestinal epithelial progenitors by enteric neurons. Proc. Natl. Acad. Sci. U. S. A. 98, 12497–12502. 10.1073/pnas.211278098 11572941 PMC60082

[B8] Bremholm L. Hornum M. Andersen U. B. Hartmann B. Holst J. J. Jeppesen P. B. (2011). The effect of Glucagon-Like Peptide-2 on mesenteric blood flow and cardiac parameters in end-jejunostomy short bowel patients. Regul. Pept. 168, 32–38. 10.1016/j.regpep.2011.03.003 21421014

[B9] Bremholm L. Hornum M. Henriksen B. M. Larsen S. Holst J. J. (2009). Glucagon-like peptide-2 increases mesenteric blood flow in humans. Scand. J. Gastroenterol. 44, 314–319. 10.1080/00365520802538195 19005872

[B10] Breslin J. W. Gaudreault N. Watson K. D. Reynoso R. Yuan S. Y. Wu M. H. (2007). Vascular endothelial growth factor-C stimulates the lymphatic pump by a VEGF receptor-3-dependent mechanism. Am. J. Physiology-Heart Circulatory Physiology 293, H709–H718. 10.1152/ajpheart.00102.2007 17400713

[B11] Brubaker P. L. Anini Y. (2003). Direct and indirect mechanisms regulating secretion of glucagon-like peptide-1 and glucagon-like peptide-2. Can. J. Physiol. Pharmacol. 81, 1005–1012. 10.1139/y03-107 14719035

[B12] Brubaker P. L. Izzo A. Hill M. Drucker D. J. (1997). Intestinal function in mice with small bowel growth induced by glucagon-like peptide-2. Am. J. Physiology-Endocrinology Metabolism 272, E1050–E1058. 10.1152/ajpendo.1997.272.6.E1050 9227451

[B13] Bulut K. Pennartz C. Felderbauer P. Meier J. J. Banasch M. Bulut D. (2008). Glucagon like peptide-2 induces intestinal restitution through VEGF release from subepithelial myofibroblasts. Eur. J. Pharmacol. 578, 279–285. 10.1016/j.ejphar.2007.08.044 17920582

[B14] Cani P. D. Possemiers S. Van de Wiele T. Guiot Y. Everard A. Rottier O. (2009). Changes in gut microbiota control inflammation in obese mice through a mechanism involving GLP-2-driven improvement of gut permeability. Gut 58, 1091–1103. 10.1136/gut.2008.165886 19240062 PMC2702831

[B15] Cazzo e. Gestic m. A. Utrini m. P. Chaim f. D. M. Geloneze b. Pareja j. C. (2016). Glp-2: a poorly understood mediator enrolled in various bariatric/metabolic surgery-related pathophysiologic mechanisms. Abcd. Arq. Bras. Cir. Dig. (são paulo) 29, 272–275. 10.1590/0102-6720201600040014 PMC5225870 28076485

[B16] Chavez–Jauregui R. N. Mattes R. D. Parks E. J. (2010). Dynamics of fat absorption and effect of sham feeding on postprandial lipema. Gastroenterology 139, 1538–1548. 10.1053/j.gastro.2010.05.002 20493191 PMC2948783

[B17] Choe K. Jang J. Y. Park I. Kim Y. Ahn S. Park D.-Y. (2015). Intravital imaging of intestinal lacteals unveils lipid drainage through contractility. J. Clin. Investigation 125, 4042–4052. 10.1172/JCI76509 PMC4639990 26436648

[B18] Cifarelli V. Appak-Baskoy S. Peche V. S. Kluzak A. Shew T. Narendran R. (2021). Visceral obesity and insulin resistance associate with CD36 deletion in lymphatic endothelial cells. Nat. Commun. 12, 3350. 10.1038/s41467-021-23808-3 34099721 PMC8184948

[B19] Cinci L. Faussone-Pellegrini M. S. Rotondo A. Mulè F. Vannucchi M. G. (2011). GLP-2 receptor expression in excitatory and inhibitory enteric neurons and its role in mouse duodenum contractility. Neurogastroenterol. Motil. 23, e383–e392. 10.1111/j.1365-2982.2011.01750.x 21752156

[B20] Dash S. Xiao C. Morgantini C. Connelly P. W. Patterson B. W. Lewis G. F. (2014). Glucagon-like peptide-2 regulates release of chylomicrons from the intestine. Gastroenterology 147, 1275–1284. 10.1053/j.gastro.2014.08.037 25173752 PMC4316201

[B21] Dash S. Xiao C. Morgantini C. Lewis G. F. (2015). New insights into the regulation of chylomicron production. Annu. Rev. Nutr. 35, 265–294. 10.1146/annurev-nutr-071714-034338 25974693

[B22] De Heer J. Pedersen J. Ørskov C. Holst J. J. (2007). The alpha cell expresses glucagon-like peptide-2 receptors and glucagon-like peptide-2 stimulates glucagon secretion from the rat pancreas. Diabetologia 50, 2135–2142. 10.1007/s00125-007-0761-6 17676310

[B23] de Heuvel E. Wallace L. Sharkey K. A. Sigalet D. L. (2012). Glucagon-like peptide 2 induces vasoactive intestinal polypeptide expression in enteric neurons via phophatidylinositol 3-kinase-γ signaling. Am. J. Physiology-Endocrinology Metabolism 303, E994–E1005. 10.1152/ajpendo.00291.2012 PMC3469609 22895780

[B24] Deniz M. Bozkurt A. Kurtel H. (2007). Mediators of glucagon-like peptide 2-induced blood flow: responses in different vascular sites. Regul. Pept. 142, 7–15. 10.1016/j.regpep.2007.01.002 17346812

[B25] Dharmsathaphorn K. Harms V. Yamashiro D. J. Hughes R. J. Binder H. J. Wright E. M. (1983). Preferential binding of vasoactive intestinal polypeptide to basolateral membrane of rat and rabbit enterocytes. J. Clin. Investigation 71, 27–35. 10.1172/JCI110748 PMC436834 6294141

[B26] Drucker D. J. Ehrlich P. Asa S. L. Brubaker P. L. (1996). Induction of intestinal epithelial proliferation by glucagon-like peptide 2. Proc. Natl. Acad. Sci. U. S. A. 93, 7911–7916. 10.1073/pnas.93.15.7911 8755576 PMC38848

[B99] Drucker D. J. (2002). Gut adaptation and the glucagon-like peptides. Gut 50, 428–435. 10.1136/gut.50.3.428 11839727 PMC1773134

[B27] Eliasson J. Hvistendahl M. K. Freund N. Bolognani F. Meyer C. Jeppesen P. B. (2021). Apraglutide, a novel glucagon-like peptide-2 analog, improves fluid absorption in patients with short bowel syndrome intestinal failure: findings from a placebo-controlled, randomized phase 2 trial. J. Parenter. Enter. Nutr. 46, 896–904. 10.1002/jpen.2223 PMC9292678 34287970

[B28] Fischer K. D. Dhanvantari S. Drucker D. J. Brubaker P. L. (1997). Intestinal growth is associated with elevated levels of glucagon-like peptide 2 in diabetic rats. Am. J. Physiology-Endocrinology Metabolism 273, E815–E820. 10.1152/ajpendo.1997.273.4.E815 9357813

[B102] Fuchs C. D. Claudel T. Mlitz V. Riva A. Menz M. Brusilovskaya K. (2023). GLP-2 improves hepatic inflammation and fibrosis in Mdr2–/– mice via activation of NR4a1/Nur77 in hepatic stellate cells and intestinal FXR signaling. Cell. Mol. Gastroenterol. Hepatol. 16, 847–856. 10.1016/j.jcmgh.2023.08.003 37572734 PMC10522987

[B103] Fuchs S. Yusta B. Baggio L. L. Varin E. M. Matthews D. Drucker D. J. (2020). Loss of Glp2r signaling activates hepatic stellate cells and exacerbates diet-induced steatohepatitis in mice. JCI Insight 23 (5), e136907. 10.1172/jci.insight.136907 PMC7205439 32191643

[B29] Geloneze B. Lima M. M. de O. Pareja J. C. Barreto M. R. L. Magro D. O. (2013). Association of insulin resistance and GLP-2 secretion in obesity: a pilot study. Arquivos Brasileiros de Endocrinol. Metabologia 57, 632–635. 10.1590/S0004-27302013000800008 24343632

[B30] Ghanem M. Lewis G. F. Xiao C. (2022). Recent advances in cytoplasmic lipid droplet metabolism in intestinal enterocyte. Biochim. Biophys. Acta Mol. Cell Biol. Lipids 1867, 159197. 10.1016/j.bbalip.2022.159197 35820577

[B31] González C. Barroso C. Martín C. Gulbenkian S. Estrada C. (1997). Neuronal nitric oxide synthase activation by vasoactive intestinal peptide in bovine cerebral arteries. J. Cereb. Blood Flow Metabolism 17, 977–984. 10.1097/00004647-199709000-00007 9307611

[B32] Grande E. M. Raka F. Hoffman S. Adeli K. (2022). GLP-2 regulation of dietary fat absorption and intestinal chylomicron production via neuronal nitric oxide synthase (nNOS) signaling. Diabetes 71, 1388–1399. 10.2337/db21-1053 35476805

[B33] Guan X. Shi X. Li X. Chang B. Wang Y. Li D. (2012). GLP-2 receptor in POMC neurons suppresses feeding behavior and gastric motility. Am. J. Physiology-Endocrinology Metabolism 303, E853–E864. 10.1152/ajpendo.00245.2012 PMC3469617 22829581

[B34] Guan X. (2014). The CNS glucagon-like peptide-2 receptor in the control of energy balance and glucose homeostasis. Am. J. Physiol. Regul. Integr. Comp. Physiol. 307, R585–R596. 10.1152/ajpregu.00096.2014 24990862 PMC4166762

[B35] Guan X. Karpen H. E. Stephens J. Bukowski J. T. Niu S. Zhang G. (2006). GLP-2 receptor localizes to enteric neurons and endocrine cells expressing vasoactive peptides and mediates increased blood flow. Gastroenterology 130, 150–164. 10.1053/j.gastro.2005.11.005 16401478

[B36] Guan X. Stoll B. Lu X. Tappenden K. A. Holst J. J. Hartmann B. (2003). GLP-2-mediated up-regulation of intestinal blood flow and glucose uptake is nitric oxide-dependent in TPN-fed piglets 1. Gastroenterology 125, 136–147. 10.1016/s0016-5085(03)00667-x 12851879

[B37] Hargrove D. M. Alagarsamy S. Croston G. Laporte R. Qi S. Srinivasan K. (2020). Pharmacological characterization of apraglutide, a novel long-acting peptidic glucagon-like peptide-2 agonist, for the treatment of short bowel syndrome. J. Pharmacol. Exp. Ther. 373, 193–203. 10.1124/jpet.119.262238 32075870

[B38] He W. Rebello O. D. Henne A. Nikolka F. Klein T. Maedler K. (2021). GLP-2 is locally produced from human islets and balances inflammation through an inter-islet-immune cell crosstalk. Front. Endocrinol. (Lausanne) 12, 697120. 10.3389/fendo.2021.697120 34290670 PMC8287580

[B39] Hein G. J. Baker C. Hsieh J. Farr S. Adeli K. (2013). GLP-1 and GLP-2 as yin and yang of intestinal lipoprotein production: evidence for predominance of GLP-2-stimulated postprandial lipemia in normal and insulin-resistant states. Diabetes 62, 373–381. 10.2337/db12-0202 23028139 PMC3554391

[B40] Higgins V. Asgari S. Hamilton J. K. Wolska A. Remaley A. T. Hartmann B. (2020). Postprandial dyslipidemia, hyperinsulinemia, and impaired gut peptides/bile acids in adolescents with obesity. J. Clin. Endocrinol. Metab. 105, 1228–1241. 10.1210/clinem/dgz261 31825485 PMC7065844

[B41] Hsieh J. Longuet C. Maida A. Bahrami J. Xu E. Baker C. L. (2009). Glucagon-like peptide-2 increases intestinal lipid absorption and chylomicron production via CD36. Gastroenterology 137, 997–1005. 10.1053/j.gastro.2009.05.051 19482026

[B42] Hsieh J. Trajcevski K. E. Farr S. L. Baker C. L. Lake E. J. Taher J. (2015). Glucagon-like peptide 2 (GLP-2) stimulates postprandial chylomicron production and postabsorptive release of intestinal triglyceride storage pools via induction of nitric oxide signaling in male hamsters and mice. Endocrinology 156, 3538–3547. 10.1210/EN.2015-1110 26132919

[B43] Hung Y.-H. Carreiro A. L. Buhman K. K. (2017). Dgat1 and Dgat2 regulate enterocyte triacylglycerol distribution and alter proteins associated with cytoplasmic lipid droplets in response to dietary fat. Biochimica Biophysica Acta (BBA) - Mol. Cell Biol. Lipids 1862, 600–614. 10.1016/j.bbalip.2017.02.014 PMC5503214 28249764

[B44] Iwai T. Jin K. Ohnuki T. Sasaki-Hamada S. Nakamura M. Saitoh A. (2015). Glucagon-like peptide-2-induced memory improvement and anxiolytic effects in mice. Neuropeptides 49, 7–14. 10.1016/j.npep.2014.11.001 25481797

[B45] Jackson K. G. Denise Robertson M. Fielding B. A. Frayn K. N. Williams C. M. (2001). Second meal effect: modified sham feeding does not provoke the release of stored triacylglycerol from a previous high-fat meal. Br. J. Nutr. 85, 149–156. 10.1079/BJN2000226 11242482

[B46] Jacome-Sosa M. Hu Q. Manrique-Acevedo C. M. Phair R. D. Parks E. J. (2021). Human intestinal lipid storage through sequential meals reveals faster dinner appearance is associated with hyperlipidemia. JCI Insight 6, e148378. 10.1172/jci.insight.148378 34369385 PMC8489663

[B47] Jeppesen P. B. Hartmann B. Thulesen J. Graff J. Lohmann J. Hansen B. S. (2001). Glucagon-like peptide 2 improves nutrient absorption and nutritional status in short-bowel patients with no colon. Gastroenterology 120, 806–815. 10.1053/gast.2001.22555 11231933

[B48] Jeppesen P. B. Lund P. Gottschalck I. B. Nielsen H. B. Holst J. J. Mortensen J. (2009). Short bowel patients treated for two years with glucagon-like peptide 2: effects on intestinal morphology and absorption, renal function, bone and body composition, and muscle function. Gastroenterol. Res. Pract. 2009, 616054–616112. 10.1155/2009/616054 19707516 PMC2729387

[B104] Kahles F. Sausen M. Quintana L. Rueckbeil M. Idel P. Mertens R. (2023). GLP-2 predicts cardiovascular outcomes in patients with myocardial infarction and increases atherosclerosis in mice. Eur. Heart J. 44 (Suppl 2), ehad655.1429. 10.1093/eurheartj/ehad655.1429

[B49] Larsen P. J. Tang-Christensen M. Holst J. J. Ørskov C. (1997). Distribution of glucagon-like peptide-1 and other preproglucagon-derived peptides in the rat hypothalamus and brainstem. Neuroscience 77, 257–270. 10.1016/S0306-4522(96)00434-4 9044391

[B50] Lewis G. F. Xiao C. Hegele R. A. (2015). Hypertriglyceridemia in the genomic era: a new paradigm. Endocr. Rev. 36, 131–147. 10.1210/er.2014-1062 25554923

[B51] Lovshin J. Estall J. Yusta B. Brown T. J. Drucker D. J. (2001). Glucagon-like peptide (GLP)-2 action in the murine central nervous system is enhanced by elimination of GLP-1 receptor signaling. J. Biol. Chem. 276, 21489–21499. 10.1074/jbc.M009382200 11262390

[B52] Lovshin J. A. Huang Q. Seaberg R. Brubaker P. L. Drucker D. J. (2004). Extrahypothalamic expression of the glucagon-like peptide-2 receptor is coupled to reduction of glutamate-induced cell death in cultured hippocampal cells. Endocrinology 145, 3495–3506. 10.1210/en.2004-0100 15059959

[B53] Meier J. J. Nauck M. A. Pott A. Heinze K. Goetze O. Bulut K. (2006). Glucagon-like peptide 2 stimulates glucagon secretion, enhances lipid absorption, and inhibits gastric acid secretion in humans. Gastroenterology 130, 44–54. 10.1053/j.gastro.2005.10.004 16401467

[B101] Murali S. G. Brinkman A. S. Solverson P. Pun W. Pintar J. E. Ney D. M. (2012). Exogenous GLP-2 and IGF-I induce a differential intestinal response in IGF binding protein-3 and -5 double knockout mice. Am. J. Physiol. Gastrointest. Liver Physiol. 302, G794–G804. 10.1152/ajpgi.00372.2011 22281475 PMC3355561

[B54] Mukherjee K. Wang R. Xiao C. (2023). Release of lipids stored in the intestine by glucagon-like peptide-2 involves a gut-brain neural pathway. Arterioscler. Thromb. Vasc. Biol. 44, 192–201. 10.1161/ATVBAHA.123.320032 37970717

[B55] Nagell C. F. Wettergren A. Pedersen J. F. Mortensen D. Holst J. J. (2004). Glucagon-like peptide-2 inhibits antral emptying in man, but is not as potent as glucagon-like peptide-1. Scand. J. Gastroenterol. 39, 353–358. 10.1080/00365520410004424 15125467

[B56] Nakame K. Kaji T. Mukai M. Shinyama S. Matsufuji H. (2016). The protective and anti-inflammatory effects of glucagon-like peptide-2 in an experimental rat model of necrotizing enterocolitis. Pept. (N.Y.) 75, 1–7. 10.1016/j.peptides.2015.07.025 26551873

[B57] Nassir F. Wilson B. Han X. Gross R. W. Abumrad N. A. (2007). CD36 is important for fatty acid and cholesterol uptake by the proximal but not distal intestine. J. Biol. Chem. 282, 19493–19501. 10.1074/jbc.M703330200 17507371

[B58] Nelson D. W. Sharp J. W. Brownfield M. S. Raybould H. E. Ney D. M. (2007). Localization and activation of glucagon-like peptide-2 receptors on vagal afferents in the rat. Endocrinology 148, 1954–1962. 10.1210/en.2006-1232 17234710

[B59] Nuzzo D. Baldassano S. Amato A. Picone P. Galizzi G. Caldara G. F. (2019). Glucagon-like peptide-2 reduces the obesity-associated inflammation in the brain. Neurobiol. Dis. 121, 296–304. 10.1016/j.nbd.2018.10.012 30347266

[B60] Ørskov C. Hartmann B. Poulsen S. S. Thulesen J. Hare K. J. Holst J. J. (2005). GLP-2 stimulates colonic growth via KGF, released by subepithelial myofibroblasts with GLP-2 receptors. Regul. Pept. 124, 105–112. 10.1016/j.regpep.2004.07.009 15544847

[B61] Pedersen J. Pedersen N. B. Brix S. W. Grunddal K. V. Rosenkilde M. M. Hartmann B. (2015). The glucagon-like peptide 2 receptor is expressed in enteric neurons and not in the epithelium of the intestine. Pept. (N.Y.) 67, 20–28. 10.1016/j.peptides.2015.02.007 25748021

[B62] Qi K. K. Wu J. Deng B. Li Y. M. Xu Z. W. (2015). PEGylated porcine glucagon-like peptide-2 improved the intestinal digestive function and prevented inflammation of weaning piglets challenged with LPS. Animal 9, 1481–1489. 10.1017/S1751731115000749 25963800

[B63] Roberge J. N. Brubaker P. L. (1991). Secretion of proglucagon-derived peptides in response to intestinal luminal nutrients. Endocrinology 128, 3169–3174. 10.1210/endo-128-6-3169 2036983

[B64] Robertson M. D. Parkes M. Warren B. F. Ferguson D. J. P. Jackson K. G. Jewell D. P. (2003). Mobilisation of enterocyte fat stores by oral glucose in humans. Gut 52, 834–839. 10.1136/gut.52.6.834 12740339 PMC1773679

[B65] Sasaki-Hamada S. Ikeda M. Oka J. I. (2019). Glucagon-like peptide-2 rescues memory impairments and neuropathological changes in a mouse model of dementia induced by the intracerebroventricular administration of streptozotocin. Sci. Rep. 9, 13723. 10.1038/s41598-019-50167-3 31548563 PMC6757030

[B105] Shew T. Wolins N. E. Cifarelli V. (2018). VEGFR-3 signaling regulates triglyceride retention and absorption in the intestine. Front. Physiol. 9, 1783. 10.3389/fphys.2018.01783 30618798 PMC6297147

[B66] Shi X. Zhou F. Li X. Chang B. Li D. Wang Y. (2013). Central GLP-2 enhances hepatic insulin sensitivity via activating PI3K signaling in POMC neurons. Cell Metab. 18, 86–98. 10.1016/j.cmet.2013.06.014 23823479 PMC3752162

[B67] Siafakas C. G. Anatolitou F. Fusunyan R. D. Walker W. A. Sanderson I. R. (1999). Vascular endothelial growth factor (VEGF) is present in human breast milk and its receptor is present on intestinal epithelial cells. Pediatr. Res. 45, 652–657. 10.1203/00006450-199905010-00007 10231859

[B68] Sigalet D. L. Wallace L. E. Holst J. J. Martin G. R. Kaji T. Tanaka H. (2007). Enteric neural pathways mediate the anti-inflammatory actions of glucagon-like peptide 2. Am. J. Physiology-Gastrointestinal Liver Physiology 293, G211–G221. 10.1152/ajpgi.00530.2006 17395898

[B69] Spessert R. (1993). Vasoactive intestinal peptide stimulation of cyclic guanosine monophosphate formation: further evidence for a role of nitric oxide synthase and cytosolic guanylate cyclase in rat pinealocytes. Endocrinology 132, 2513–2517. 10.1210/endo.132.6.7684978 7684978

[B70] Stahel P. Xiao C. Davis X. Tso P. Lewis G. F. (2019). Glucose and GLP-2 (Glucagon-Like peptide-2) mobilize intestinal triglyceride by distinct mechanisms. Arterioscler. Thromb. Vasc. Biol. 39, 1565–1573. 10.1161/ATVBAHA.119.313011 31294621 PMC6657524

[B71] Stahel P. Xiao C. Hegele R. A. Lewis G. F. (2018). The atherogenic dyslipidemia complex and novel approaches to cardiovascular disease prevention in diabetes. Can. J. Cardiol. 34, 595–604. 10.1016/j.cjca.2017.12.007 29459241

[B72] Stahel P. Xiao C. Nahmias A. Lewis G. F. (2020). Role of the gut in diabetic dyslipidemia. Front. Endocrinol. (Lausanne) 11, 116. 10.3389/fendo.2020.00116 32231641 PMC7083132

[B98] Stahel P. Xiao C. Nahmias A. Tian L. Lewis G. F. (2021). Multi-organ coordination of lipoprotein secretion by hormones, nutrients and neural networks. Endocr. Rev. 42, 815–838. 10.1210/endrev/bnab008 33743013 PMC8599201

[B73] Su Y. Zhang Z. Li H. Ma J. Sun L. Shao S. (2021). A GLP-2 analogue protects SH-SY5Y and neuro-2a cells against mitochondrial damage, autophagy impairments and apoptosis in a Parkinson model. Drug Res. 71, 43–50. 10.1055/a-1266-3263 33022720

[B74] Sun H. Meng K. Hou L. Shang L. Yan J. (2021). Melanocortin receptor-4 mediates the anorectic effect induced by the nucleus tractus solitarius injection of glucagon-like Peptide-2 in fasted rats. Eur. J. Pharmacol. 901, 174072. 10.1016/j.ejphar.2021.174072 33823184

[B75] Syed-Abdul M. M. Stahel P. Tian L. Xiao C. Nahmias A. Lewis G. F. (2022a). Glucagon-like peptide-2 mobilization of intestinal lipid does not require canonical enterocyte chylomicron synthetic machinery. Biochimica Biophysica Acta (BBA) - Mol. Cell Biol. Lipids 1867, 159194. 10.1016/j.bbalip.2022.159194 35680083

[B76] Syed-Abdul M. M. Tian L. Xiao C. Lewis G. F. (2022b). Lymphatics - not just a chylomicron conduit. Curr. Opin. Lipidol. 33, 175–184. 10.1097/MOL.0000000000000821 35258031

[B77] Taher J. Baker C. Alvares D. Ijaz L. Hussain M. Adeli K. (2018). GLP-2 dysregulates hepatic lipoprotein metabolism, inducing fatty liver and VLDL overproduction in male hamsters and mice. Endocrinology 159, 3340–3350. 10.1210/en.2018-00416 30052880

[B78] Talbot J. Hahn P. Kroehling L. Nguyen H. Li D. Littman D. R. (2020). Feeding-dependent VIP neuron–ILC3 circuit regulates the intestinal barrier. Nature 579, 575–580. 10.1038/s41586-020-2039-9 32050257 PMC7135938

[B79] Tang-Christensen M. Larsen P. J. Thulesen J. Rømer J. Vrang N. (2000). The proglucagon-derived peptide, glucagon-like peptide-2, is a neurotransmitter involved in the regulation of food intake. Nat. Med. 6, 802–807. 10.1038/77535 10888930

[B80] Velázquez E. Le Baut Ayuso Y. Blázquez E. Ruiz-Albusac J. M. (2022). Glucose and several mitogenic agents modulate the glucagon-like peptide-2 receptor expression in cultured rat astrocytes. J. Alzheimers Dis. Rep. 6, 723–732. 10.3233/ADR-220043 36606205 PMC9741749

[B81] Velázquez E. Ruiz‐Albusac J. M. Blázquez E. (2003). Glucagon-like peptide-2 stimulates the proliferation of cultured rat astrocytes. Eur. J. Biochem. 270, 3001–3009. 10.1046/j.1432-1033.2003.03677.x 12846833

[B82] Verdam F. J. Greve J. W. M. Roosta S. van Eijk H. Bouvy N. Buurman W. A. (2011). Small intestinal alterations in severely obese hyperglycemic subjects. J. Clin. Endocrinol. Metab. 96, E379–E383. 10.1210/jc.2010-1333 21084402

[B83] Vrang N. Hansen M. Larsen P. J. Tang-Christensen M. (2007). Characterization of brainstem preproglucagon projections to the paraventricular and dorsomedial hypothalamic nuclei. Brain Res. 1149, 118–126. 10.1016/j.brainres.2007.02.043 17433266

[B84] Vrang N. Larsen P. J. Jensen P. B. Lykkegaard K. Artmann A. Larsen L. K. (2008). Upregulation of the brainstem preproglucagon system in the obese Zucker rat. Brain Res. 1187, 116–124. 10.1016/j.brainres.2007.10.026 18022140

[B85] Wismann P. Barkholt P. Secher T. Vrang N. Hansen H. B. Jeppesen P. B. (2017). The endogenous preproglucagon system is not essential for gut growth homeostasis in mice. Mol. Metab. 6, 681–692. 10.1016/j.molmet.2017.04.007 28702324 PMC5485241

[B86] Wøjdemann M. Wettergren A. Hartmann B. Holst J. J. (1998). Glucagon-like peptide-2 inhibits centrally induced antral motility in pigs. Scand. J. Gastroenterol. 33, 828–832. 10.1080/00365529850171486 9754730

[B97] Xiao C. Dash S. Morgantini C. Adeli K. Lewis G. F. (2015). Gut peptides are novel regulators of intestinal lipoprotein secretion: experimental and pharmacological manipulation of lipoprotein metabolism. Diabetes 64, 2310–2318. 10.2337/db14-1706 26106188

[B87] Xiao C. Stahel P. Carreiro A. L. Buhman K. K. Lewis G. F. (2018). Recent advances in triacylglycerol mobilization by the gut. Trends Endocrinol. Metabolism 29, 151–163. 10.1016/j.tem.2017.12.001 29306629

[B88] Xiao C. Stahel P. Carreiro A. L. Hung Y.-H. Dash S. Bookman I. (2019a). Oral glucose mobilizes triglyceride stores from the human intestine. Cell Mol. Gastroenterol. Hepatol. 7, 313–337. 10.1016/j.jcmgh.2018.10.002 30704982 PMC6357697

[B89] Xiao C. Stahel P. Lewis G. F. (2019b). Regulation of chylomicron secretion: focus on post-assembly mechanisms. Cell Mol. Gastroenterol. Hepatol. 7, 487–501. 10.1016/j.jcmgh.2018.10.015 30819663 PMC6396431

[B90] Xiao C. Stahel P. Morgantini C. Nahmias A. Dash S. Lewis G. F. (2019c). Glucagon‐like peptide‐2 mobilizes lipids from the intestine by a systemic nitric oxide‐independent mechanism. Diabetes Obes. Metab. 21, 2535–2541. 10.1111/dom.13839 31364232

[B91] Xiao C. Stahel P. Nahmias A. Lewis G. F. (2020). Emerging role of lymphatics in the regulation of intestinal lipid mobilization. Front. Physiol. 10, 1604. 10.3389/fphys.2019.01604 32063861 PMC7000543

[B92] Yusta B. Huang L. Munroe D. Wolff G. Fantaske R. Sharma S. (2000). Enteroendocrine localization of GLP-2 receptor expression in humans and rodents. Gastroenterology 119, 744–755. 10.1053/gast.2000.16489 10982769

[B100] Yusta B. Holland D. Koehler J. A. Maziarz M. Estall J. L. Higgins R. (2009). ErbB signaling is required for the proliferative actions of GLP-2 in the murine gut. Gastroenterology 137, 986–996. 10.1053/j.gastro.2009.05.057 19523469

[B93] Yusta B. Matthews D. Koehler J. A. Pujadas G. Kaur K. D. Drucker D. J. (2019). Localization of glucagon-like peptide-2 receptor expression in the mouse. Endocrinology 160, 1950–1963. 10.1210/en.2019-00398 31237617 PMC6656427

[B94] Zembroski A. S. D’Aquila T. Buhman K. K. (2021). Characterization of cytoplasmic lipid droplets in each region of the small intestine of lean and diet-induced obese mice in response to dietary fat. Am. J. Physiol. Gastrointest. Liver Physiol. 321, G75–G86. 10.1152/AJPGI.00084.2021 34009042 PMC8321799

[B95] Zhang F. Zarkada G. Han J. Li J. Dubrac A. Ola R. (2018). Lacteal junction zippering protects against diet-induced obesity. Science 361, 599–603. 10.1126/science.aap9331 30093598 PMC6317738

[B96] Zhang Z. Hao L. Shi M. Yu Z. Shao S. Yuan Y. (2021). Neuroprotective effects of a GLP-2 analogue in the MPTP Parkinson’s disease mouse model. J. Park. Dis. 11, 529–543. 10.3233/JPD-202318 33523018

